# Cosegregation of congenital dysferlinopathy phenotype and marinesco–sjögren syndrome: a case report with literature review

**DOI:** 10.1186/s12887-026-06651-7

**Published:** 2026-03-05

**Authors:** Sergey N. Bardakov, Alexey M. Emelin, Sergey S. Nikitin, Vasiliy М. Suslov, Ivan V. Dmitrochenko, Irina S. Kovalevskaya, Polina R. Korzun, Ivan A. Yakovlev, Аrtur А. Isaev, Roman V. Deev

**Affiliations:** 1 Department of Neurology, S.M. Kirov Military Medical Academy, 6 Lebedeva St., St. Petersburg, 194044 Russia; 2 A.P. Avtsyn Research Institute of Human Morphology of Federal state budgetary scientific institution “Petrovsky National Research Centre of Surgery“, Ministry of Science and Higher Education of Russia, 3 Tsyurupy St, Moscow, 117418 Russia; 3 Research Centre for Medical Genetics, 1 Moskvorechye St, Moscow, 115522 Russia; 4 Saint-Petersburg State Pediatric Medical University, 2 Litovskaya St, St. Petersburg, 194100 Russia; 5 Artgen Biotech PJSC, 3 Gubkina St, Moscow, 119333 Russia; 6 Genotarget LLC, Skolkovo Innovation Centre, Moscow, 121614 Russia; 7 Genetico, 3 Gubkina St., Bldg. 1, Moscow, 119333 Russia

**Keywords:** dysferlinopathy, limb-girdle muscular dystrophy R2, congenital phenotype, DYSF, Marinesco–Sjögren syndrome, myopathy

## Abstract

The congenital dysferlinopathy phenotype is the rarest and earliest manifestation variant, described in two closely related Spanish and Turkish families, with a homozygous pathogenic frameshift variant in exon 26 of the *DYSF* gene.

This article presents a 1.6-year-old patient from a consanguineous Uzbek family with a clinical diagnosis of congenital dysferlinopathy phenotype and Marinesco**–**Sjögren syndrome with transient postnatal hypotonia, motor development delay, muscle weakness in the flexors of the neck and proximal limbs, convergent strabismus, cerebellar truncal ataxia, minimal intention tremor of the upper limbs, a slight increase in the levels of creatinine phosphokinase (CK) to 353 U/L (2.4×N) and that of myoglobin to 40 µg/mL (2×N).

Magnetic resonance imaging (MRI) revealed pronounced edematous changes in the gastrocnemius muscle on short tau inversion recovery (STIR). MRI signs of minimal fat replacement and hypotrophy were noted in the medial and posterior thigh and gastrocnemius muscles. MRI of the head revealed hypoplasia of the vermis and cerebellar hemispheres. Whole-exome sequencing revealed compound heterozygous *DYSF* variants: NM_001130987.2:c.1000 C > T (p.(Arg334Trp)) and NM_001130987.2:c.518 C > T (p.(Thr173Met)), and a previously described homozygous *SIL1* variant NM_022464.5:c.178G > T (p.(Glu60Ter)). Histopathological examination revealed minimal signs of myopathy and dysferlin in only 1% of the muscle fibers. At the ultrastructural level, signs of dysferlinopathy and Marinesco–Sjögren syndrome were detected.

This clinical case is an example of the cosegregation of two diseases that mutually potentiate damage to skeletal muscles.

## Introduction

Dysferlinopathy is an autosomal recessive, clinically variable, progressive hereditary muscular dystrophy caused by variants in the *DYSF* gene (OMIM# 603009) [1, 2].

In the course of the disease, a pre-manifest stage is distinguished, including asymptomatic (isolated hyperCKemia) and oligosymptomatic forms, as well as a manifest stage realized in the form of distal Miyoshi myopathy, a limb-girdle phenotype, and a proximodistal form [3, 4]. On average, the onset of the manifest stage occurs between the ages of 15 and 27 years [5–7]. However, Paradas et al. (2008) [8] and Ceyhan-Birsoy et al. (2015) [9] described extremely rare cases of the congenital dysferlinopathy phenotype in Spanish (2 and 5 years) and Turkish (14 and 11 years) children caused by a homozygous *DYSF* variant in exon 26, leading to a reading-frame shift. In all cases, postnatal hypotonia; delay in all stages of motor development (onset of walking from 19 to 30 to months); weakness of the muscles of the neck, pelvic girdle, and hips; and loss of walking at 8 years were observed in one case. The CK level was 200–241 U/L, increasing after 2 years to 700–1200 U/L. A characteristic MRI sign was an increase in the signal intensity on STIR from the calf muscles and the posterior group of thigh muscles, indicating edematous changes.

Marinesco–Sjögren syndrome (MSS; OMIM #248800) is an autosomal recessive multisystem disorder characterized by psychomotor retardation, microcephaly, strabismus, early cataracts, nystagmus, cerebellar ataxia, and myopathic syndrome [10]. Patients may also develop hypergonadotropic hypogonadism, dysarthria, short stature, skeletal abnormalities, deafness, epilepsy, or Parkinson’s syndrome [11]. MSS is caused by variants in the nucleotide exchange factor gene *SIL1*, mapped to locus 5q31 [11, 12].

In this report, we present the case of a 1.6-year-old patient from a consanguineous family with a combination of congenital dysferlinopathy phenotype and MSS, mutually potentiating skeletal muscle damage.

The aim of the study is to present the clinical, instrumental, and pathomorphological features of the combination of congenital dysferlinopathy and MSS.

## Materials and methods

### Ethics statement

All the studies were conducted after the patients’ parents signed a voluntary informed consent form in accordance with the requirements of the 2013 Helsinki Declaration and the local ethics committee of the Military Medical Academy S.M. Kirov (Russia) (protocol # 219, 12/02/2020). The proband examined was a 1 year 6-month-old boy from a consanguineous Uzbek family (first cousins). Clinical and neurological examinations and genealogical analyses of the proband’s family were conducted.

### Laboratory and instrumental research

The proband underwent clinical and biochemical blood tests, stimulation, needle electromyography (EMG), electrocardiography (ECG), echocardiography, and whole-body magnetic resonance imaging (MRI).

MR scanning of the limb muscles was performed using an MR tomograph (Philips Ingenia, 1.5 T) with a surface coil. The protocol included T1 and STIR pulse sequences in three standard mutually perpendicular planes, with 30 slices and a slice thickness of 7 mm. The changes identified were assessed using the Mercuri scale (2002).

### Genetic research

A venous blood DNA sample obtained from the proband was analyzed using whole exome sequencing (next generation sequencing). Sequencing was performed using the paired-end method (2 × 75 bp) on an Illumina NextSeq 500 platform using the selective capture technique for DNA regions related to the coding regions of > 20,000 genes (Illumina TruSeq ExomeKit). The average read coverage for whole exome sequencing in all samples was 80.7×, with a read length of 2 × 75 bp. For whole- exome sequencing, the average read coverage was 44.9×, with a read length of 2 × 151 bp. To estimate the population frequencies of the identified variants, samples from the 1000 Genomes Project, ESP6500, and Genome Aggregation Database were used. PolyPhen, SIFT (Sorting Intolerant From Tolerant), and MutationTaster programs were used to predict the possible effects of amino acid substitutions and protein functions.

Potentially pathogenic variants were identified by comparison to the normal human genome, and the detected changes were confirmed using reference methods (Sanger sequencing and PCR) in the proband and his parents. Detection of exon 7 deletions in the SMN1 gene was performed using PCR.

Cytogenetic research was performed using a standard technique.

### Histopathological examination

Paraffin sections of the m. vastus lateralis biopsy specimen (right) of the proband were stained with hematoxylin and eosin (H&E), Van Gieson, and immunohistochemically with antibodies against dysferlin (ab124684, Abcam, UK), CD3, CD4, CD8, CD68, CD138, and HLA-DR (Abcam, UK). Muscle fiber typing was performed with myosin heavy chains using antibodies against MYH-1 (fast) (ab127539, Abcam, UK) and anti-myosin skeletal (slow) (m8481, Merck, USA). A biopsy specimen of the m. vastus lateralis of a healthy 3-year-old child was used as the control. A part of the biopsy specimen was processed for transmission electron microscopy using a standard technique.

### Case presentation

The proband, aged 1 year 6 months, was born from the 3rd pregnancy at 39 weeks without perinatal pathology to clinically healthy Uzbek parents. The Apgar score was 8–8, weight was 3510 g, and length was 51 cm. At birth, slight hypotension was noted for 2–3 months. The siblings (10 and 7 years old, female) were clinically healthy. Family history was characterized by the absence of neuromuscular diseases and CNS pathology.

At the time of examination, the proband was characterized by dyshormonal development: normal body length of 81 cm (z-score: − 1.77; percentile: 3.8), low body weight of 9 kg (z-score: − 2.38; percentile: 0.9), decreased weight-for-length (z-score: − 2.07; percentile: 1.9), decreased weight-for-age (z-score: − 2.38; percentile: 0.9), body mass index of 13.7, and head circumference of 45 cm (2nd percentile). Delayed motor development was noted: crawls since 9 months, does not sit independently, stands with support since 1.7 months, and cannot stand or walk independently.

**Fig. 1:**
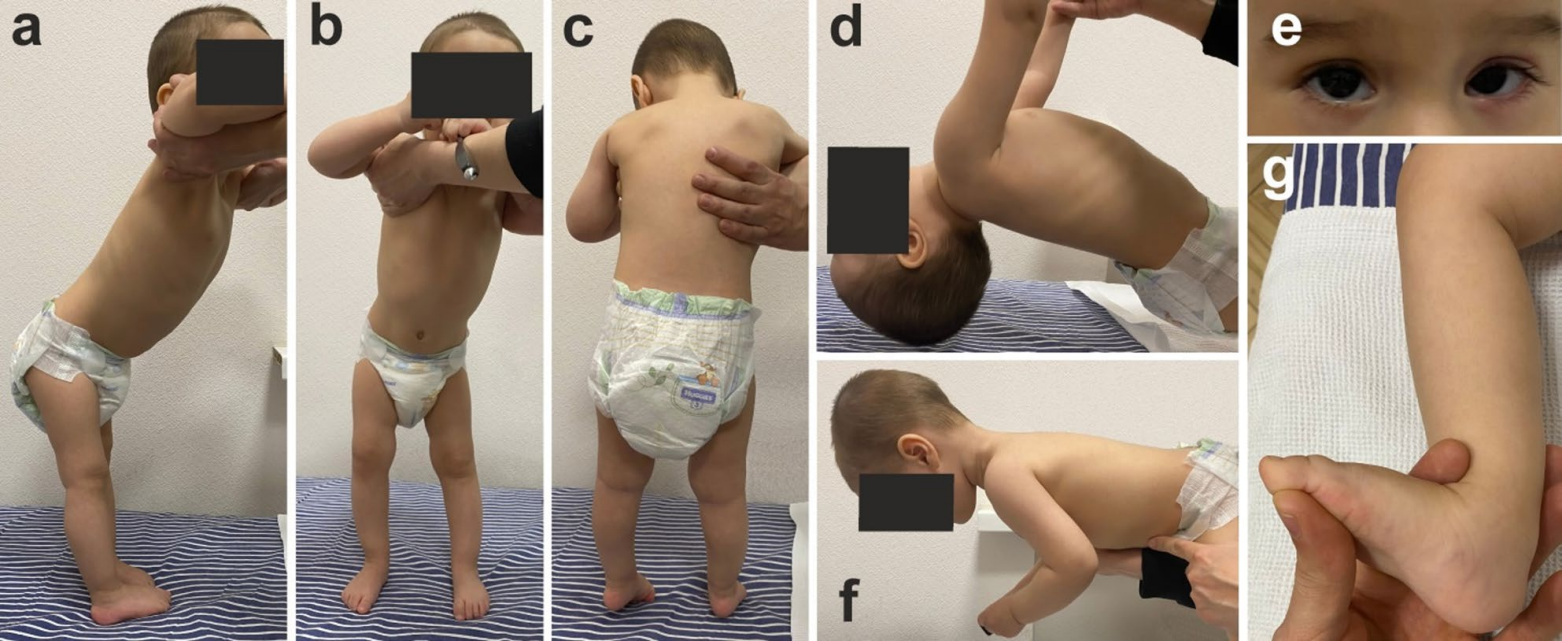
Clinical phenotype of a 1.9-year-old proband. **a**, **b**: with vertical fixation, the legs are straightened at the knees, and support is mainly on the toes with bent toes; **c**: signs of mild hypotrophy of the calf muscles; **d**: traction on the arms is accompanied by extension of the head due to muscle weakness of the neck flexors; **f**: horizontal suspension is accompanied by holding the head in an extension position and flexion of the arms and legs; **e**: convergent paternal strabismus with a vertical and accommodative component; **g**: absence of contractures of the Achilles tendons

Neurological examination revealed satisfactory muscle tone. When performing arm traction, a delay in head flexion (due to weakness of the neck flexors) is noted. The postural reactions in the vertical and horizontal suspensions were normal. Muscle strength was reduced in the neck flexors by 2 points; deltoid muscles, 4 points; and flexors and adductors of the thighs, 4 points. Mild symmetrical hypotrophy of the gastrocnemius muscles was observed. The reflexes of the upper and lower extremities were moderate. Trunk ataxia (swaying of the trunk in the sitting position), minimal intention tremor, and mild dysmetria of the upper extremities were observed. Pathological pyramidal signs were not observed. Convergent paretic strabismus with vertical and accommodative components (limitation of lateral movement OD 2nd degree, OS 3rd degree), and mild nystagmus at extreme abduction of the eyeballs were observed, along with moderate hyperopia in both eyes (Fig. 1).

### Laboratory and instrumental research

At the age of 1 year 6 months, at the time of the initial examination, an increase in the level of serum CK to 353 U/L (normal, 30–150 U/L) [13] and myoglobin to 40 µg/L (0,0–25 µg/L) was noted [14], with normal levels of ALT, ​17 U/L (11,0–25,0 U/L) [15]; AST, 34 U/L (0,0–59,0 U/L) [15]; creatinine, 18 µmol/L (27–42 µmol/L) [14]; and LDH, 329 U/L (0,0–344,0 U/L) [16]; thyroid-stimulating hormone, parathyroid hormone, adrenocorticotropic hormone, follicle-stimulating hormone, and luteinizing hormone levels were normal.

Electromyographic examination did not reveal any disturbances in nerve conduction velocity. Needle EMG revealed changes in the motor unit action potential (MUAP) characteristics of primary muscular damage.

No pathology was observed during ECG and EchoCG.

**Fig. 2:**
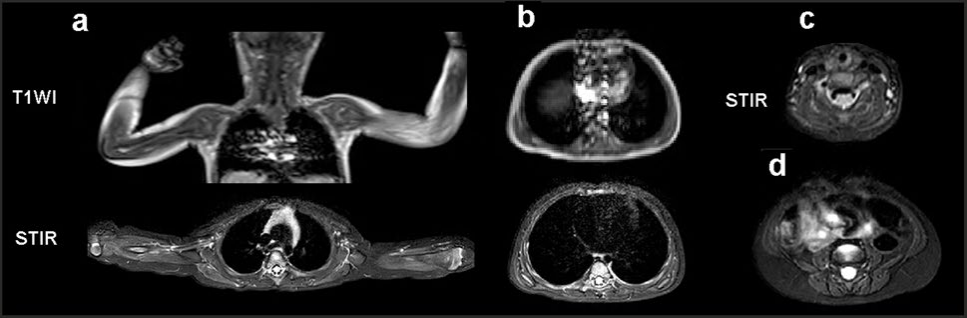
MRI of the shoulder girdle muscles of a 1.8-month-old proband (T1, STIR). **a**: shoulder girdle muscles (T1, STIR); **b**: trunk muscles at the level of Th6-7 (T1, STIR); **c**: neck muscles (STIR); and **d**: trunk muscles at the level of L5 (STIR)

**Fig. 3:**
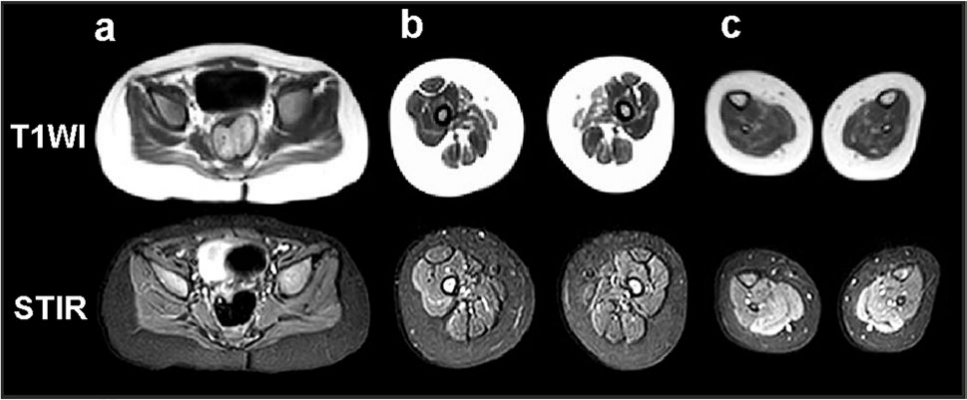
MRI (T1, STIR) of the pelvic muscles (**a**), thigh muscles (**b**), and legs (**c**)

MRI of the whole body revealed a symmetrical diffuse minimal increase in the MR signal on STIR from the m. infraspinatus, a pronounced increase from the m. soleus, and both heads of the m. gastrocnemius due to moderate edematous changes (Figs. 2 and 3).

MRI signs of minimally expressed symmetrical fat replacement (hyperintense on T1 and hypointense on STIR), corresponding to stage 1 according to Mercuri, were observed in m. gluteus maximus, long and short heads of m. biceps femoris, m. semitendinosus, m. abductor magnus, m. soleus, and both heads of the m. gastrocnemius. Symmetrical minimally expressed hypotrophy of the m. gluteus maximus and m. abductor magnus, and asymmetric hypotrophy of the m. gastrocnemius, mainly on the left (cross-sectional area in the middle third 1.27 and 0.63 cm^2^) were revealed (Fig. 3).

### Brain MRI

In the structure of the vermis and cerebellar peduncles, irregularly shaped areas of ​​pathological intensity with unclear contours, homogeneity, and weak hyperintensity on T2 and FLAIR signal characteristics were determined. The width of the cerebellar cortex is diffusely reduced owing to atrophy. In the periventricular white matter of the parietal-occipital regions, small, irregularly shaped areas with unclear contours that were weakly hyperintense on T2 and FLAIR of a non-specific nature (reduced myelination as a manifestation of immaturity) were determined.

**Fig. 4:**
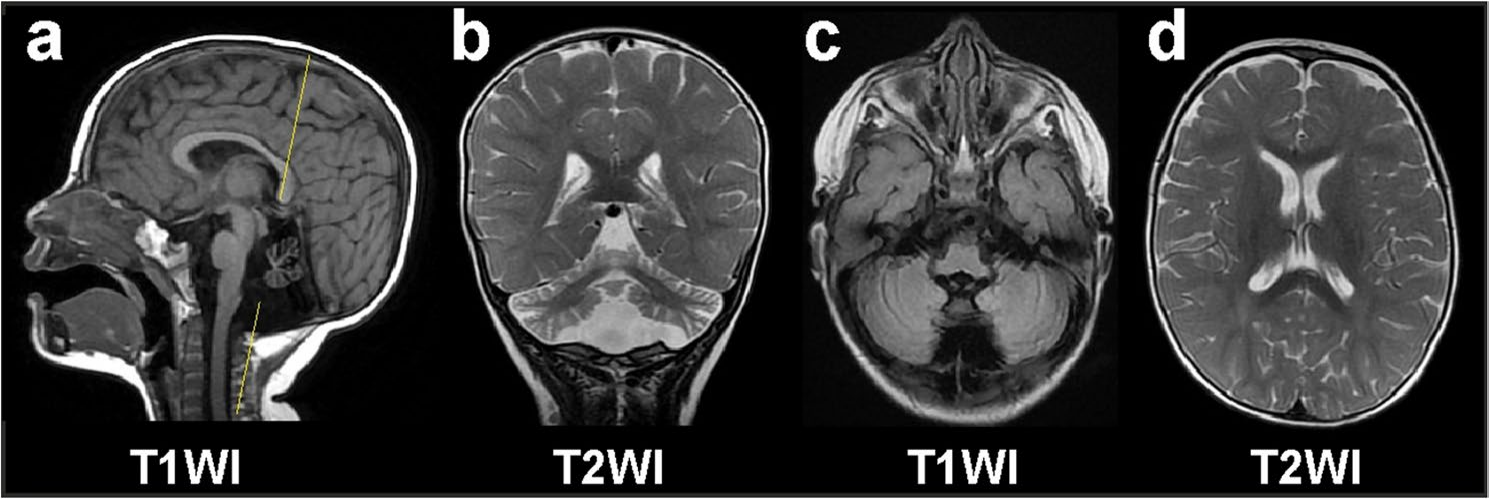
MRI of the head of a 1.8-year-old proband. **a** hypoplasia of the cerebellar vermis (sagittal plane); **b**, **c**: hypoplasia of the cerebellar hemispheres, thinning of the cerebellar cortex; and d: parieto-occipital periventricular areas of decreased myelination

Signs of encephalomalacia of the vermis and cerebellar peduncles, cerebellar atrophy, and small areas of gliosis in the periventricular white matter in the posterior horns of the lateral ventricles were observed on MR (Fig. 4).

### Genetic research

Cytogenetic study: karyotype 46,XY (male).

Whole-exome sequencing of the proband’s DNA sample revealed a previously described likely pathogenic variant in a homozygous state in exon 3 of *SIL1* gene: NM_022464.5:c.178G > T (p.(Glu60Ter)), leading to a premature termination codon. Variant annotation is provided on the GRCh38 reference genome unless otherwise stated. The *SIL1* variant corresponds to GRCh38: chr5:139121101 C > A. The variant was classified as likely pathogenic (LP) according to the ACMG/AMP criteria (PVS1, PM2_Supporting, PP4), as it represents a loss-of-function variant in a gene where loss of function is a known disease mechanism, is extremely rare in population databases, and was identified in the homozygous state in a patient with a clinical phenotype consistent with Marinesco–Sjögren syndrome. This variant has not been reported in the gnomAD v4.1.0 (access date: 09/02/26). database and has been previously described in patients with Marinesco–Sjögren syndrome [11].

A heterozygous variant was detected in the *DYSF* gene in exon 11 (NM_001130987.2:c.1000 C > T (р.Arg334Trp); GRCh38: chr2:71517037 C > T). This variant is registered in ClinVar as a variant of uncertain significance (Variation ID: 1360207) and has been reported in the literature [17]. The variant was registered in the control samples as “1000 genomes” 0.0002, and gnomAD 0.00002. According to the SpliceAI prediction algorithm, the variant may affect the consensus splicing site. Algorithms developed to predict the effect of missense changes on protein structure and function are either unavailable or do not agree on the potential impact of this missense change (SIFT: “Deleterious”; PolyPhen-2: “Probably Damaging”; Align-GVGD: “Class C0”). According to ACMG criteria, this variant is a variant of uncertain significance (VUS) based on its very low frequency in population databases (PM2_Supporting) and supportive in silico predictions suggesting a possible deleterious effect (PP3).

However, due to the lack of functional studies and the absence of extended disease segregation data, the available evidence is insufficient to support pathogenicity.

Also, a heterozygous variant was identified in the *DYSF* gene in exon 6 (NM_001130987.2:c.518 C > T (р.(Thr173Met)); GRCh38: chr2: 71513297 C > T). This variant is registered in ClinVar as a variant of uncertain significance (Variation ID: 4526590). The variant was registered as gnomAD (0.0000455). According to the ACMG criteria, the variant was assessed as a variant of uncertain significance (VUS). Parental testing confirmed that the two DYSF variants are in trans (compound heterozygous). The *SIL1* variant was identified in a homozygous state in the proband; however, parental testing for *SIL1* was not performed. The identified genotypes are consistent with autosomal recessive inheritance. The identified variants were confirmed using Sanger sequencing (Table 1).

**Table 1:** Variants identified in a 1.8-year-old proband using whole exome sequencing

Gene	Position (GRCh38)	Position in cDNA	Genotype	Classification	Carrier	Segregation
*DYSF*	Chr2:71517037 C > T	с.1000С > T (р.(Arg334Trp))	С/T	Variant of uncertain significance (VUS)	Proband	Mother
*DYSF*	Chr2:71513297 C > T	с.518С > T (р.(Thr173Met))	C/T	Variant of uncertain significance (VUS)	Proband	Father
*SIL1*	Chr5:139121101 C > A	c.178G > T (p.(Glu60Ter))	A/A	Likely pathogenic (LP)	Proband	Parental testing not performed)

No deletions of exon 7 in the *SMN* 1 gene were detected.

### Histopathological examination

**Fig. 5:**
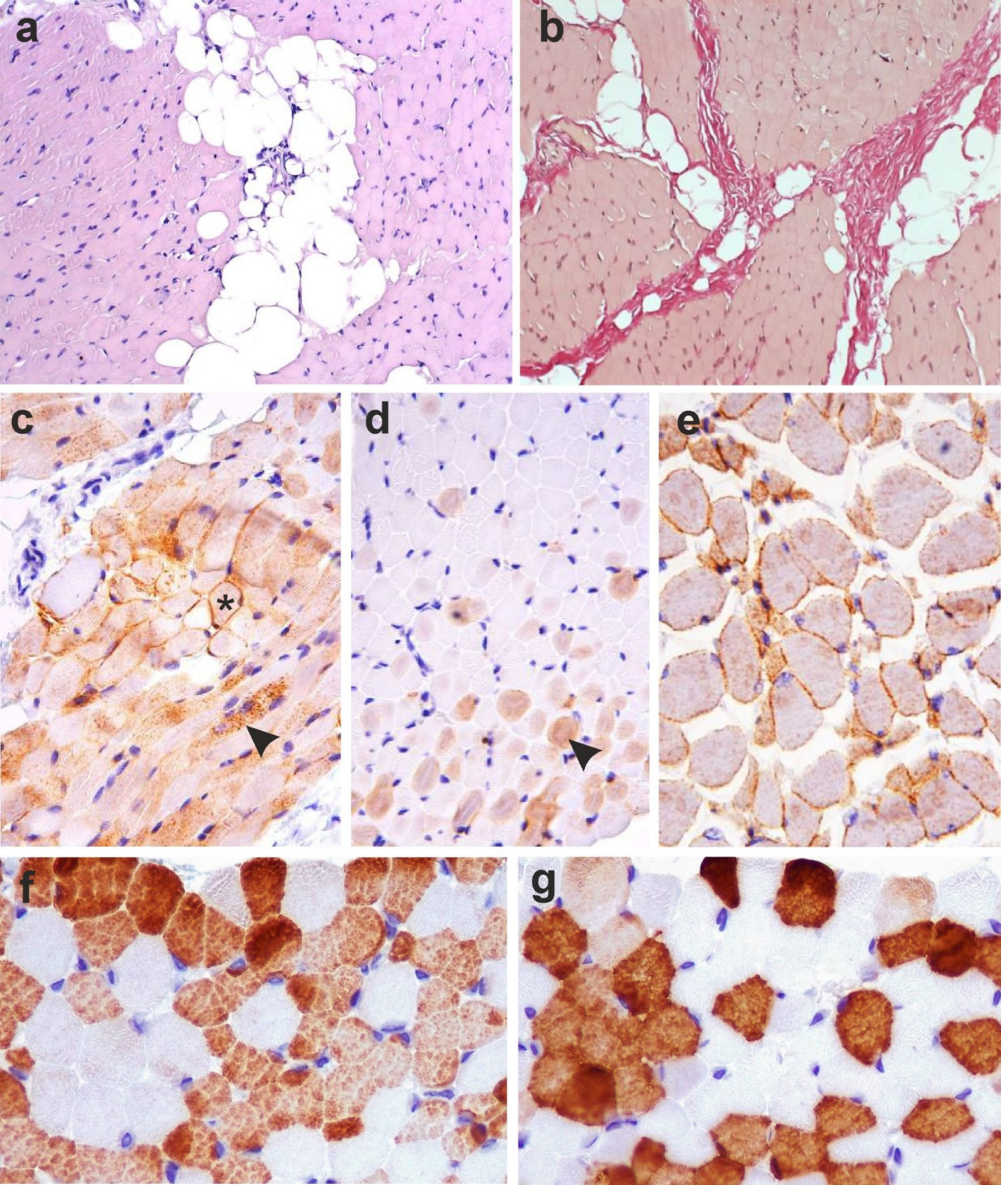
Cross-section of a skeletal muscle fragment. **a** areas of lipomatosis in the endomysium and perivascular zones of the perimysium. Hematoxylin and eosin staining. Magnification ×200; **b**: pronounced fibrosis of the endomysium and perimysium. Van Gieson staining. Magnification ×200. Immunohistochemical reaction with antibodies against dysferlin. Membrane and sarcoplasmic localization in < 1% of muscle fibers; **c**: membrane staining pattern (*); vesicular staining pattern (arrow); **d**: sarcoplasmic staining pattern due to non-specific deposition of chromogen; **e**: positive control, membrane pattern of the immunohistochemical reaction. Counterstaining with hematoxylin. Magnification ×400; typing of muscle fibers using myosin heavy chains; **f**: immunohistochemical (IHC) reaction with antibodies against MYH-1 (skeletal, fast), normal amount of fast MV (54 (49; 61)%); **g**: IHC reaction with antibodies against myosin (skeletal, slow), normal ratio of slow muscle fibers (44 (39; 50)%). Magnification ×400

In the biopsy of the m. vastus lateralis (right), a minimally expressed myopathic pattern of changes was revealed in the form of a small number of rounded atrophic fibers, single necrotic fibers, areas of pronounced lipomatosis in the endomysium and perivascular zones of the perimysium, and moderately expressed fibrosis in the endomysium (Fig. 5a, b). ​ Immunohistochemical examination of the lymphocyte–macrophage infiltrate composition confirmed the presence of single CD3 + and CD8 + lymphocytes in the perimysium, and single macrophages (CD68+) near the necrotic muscle fibers, indicating the absence of significant inflammation. According to the immunohistochemical reaction with antibodies (clone JAI-1-49-3), dysferlin was not reliably detected in the predominant part of the muscle fibers; a positive reaction was detected in < 1% of the muscle fibers in the form of membrane and cytoplasmic (non-specific) staining patterns (Fig. 5c, d, e). When typing muscle fibers using antibodies to myosin heavy chains, no disturbance in the ratio of muscle fiber types was found (44 (39; 50)% slow fibers; 54 (49; 61)% fast fibers) [18] (Fig. 5f, g).

At the ultrastructural level, uneven expansion of the basement membrane of muscle fibers with areas of integrity disruption and thickening were observed. The sarcolemma had scalloped areas with partial integrity disruption, with accumulation of vesicles in these areas and expansion of subsarcolemmal spaces that contained flocculent detritus and single-membrane myelin-like inclusions. Myofibrils were dissociated and had small focal lysis. The structure of sarcomeres was disrupted, and the Z-line in most sarcomeres was either absent or weakly expressed. The cellular organelles were destructured.

Pronounced destructive changes in nuclei was observed, with partial absence of nucleolemma, clearing of nucleoplasm with fine lumpy contents, and abnormal chromatin aggregation. Nuclei with perinuclear vacuoles were observed in individual fibers.

Notably, the subsarcolemmal and interfibrillar clusters of polymorphic small mitochondria showed disruption of membrane integrity and destruction of the cristae.

**Fig. 6:**
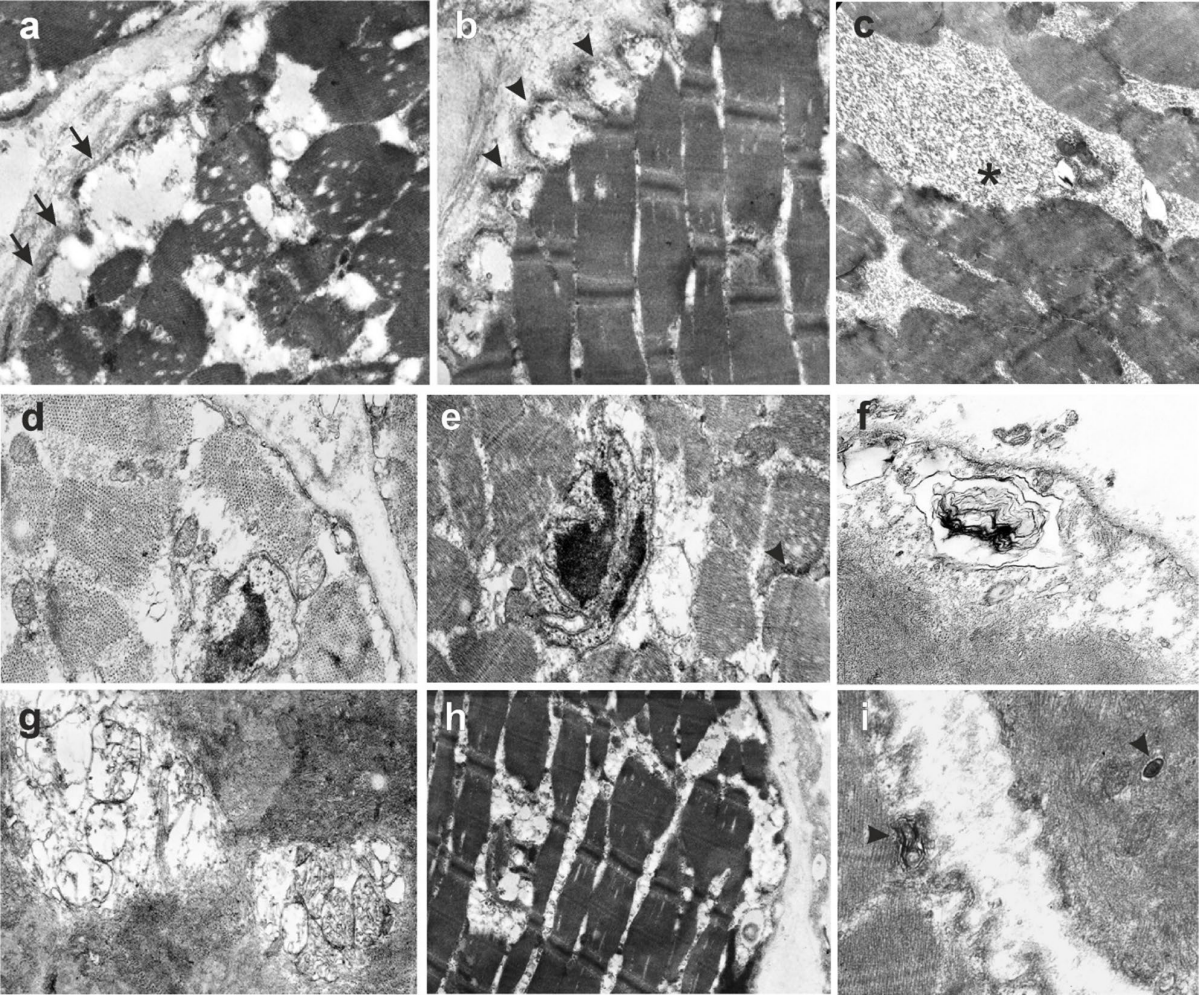
Ultrastructural changes in muscle fibers. **a** Pronounced destructive changes in the muscle fiber. The structure of sarcomeres is disrupted; the Z-line is absent or weakly expressed in most sarcomeres. The subsarcolemmal space is expanded, of low electron density with flocculent contents. Dissociation of myofibrils; destruction of cellular organelles. Small focal lysis of myofibrils. The basement membrane of the muscle fiber is swollen and fragmented. The plasma membrane is present in fragments (arrows). Decreased or absent glycogen; absence of the T-system. Magnification ×17,000. **b** Scalloped edge of muscle fiber. In places where plasma membrane is absent, the accumulation of vesicles is observed (as a manifestation of muscle fiber regeneration). Disorganization of Z-lines. Small focal lysis of myofibrils. Magnification ×14000. **c** Accumulation of osmiophilic granular material in the sarcoplasm. Magnification ×23000. **d** Cross-section of destructively altered muscle fibers. The integrity of the plasma membrane is disrupted. Myofibrils were then disunited. Destructive changes in mitochondria occurred in the form of focal loss of the double contour of the outer membrane and destruction in certain areas. Pronounced destructive changes in the nuclei include a dense membranous structure around the nucleus, partial absence of the nucleolemma, clearing of the nucleoplasm with fine lumpy contents, and abnormal chromatin aggregation. Edema of the sarcoplasm with single dilated T-system tubules. Magnification ×23000. **e** Fragments of destructively altered muscle fibers. Edema of the sarcoplasm with fine granular content. Osmiophilic content of the dilated sarcoplasmic reticulum canaliculus. Myofibrils were separated by lysis foci, and Z-lines were not determined. Single T-tubules (arrows). Nuclei with multiple invaginations and partial absence of nucleolemma, nucleoplasm with lumpy contents, and abnormal chromatin aggregation. Magnification ×17,000. **f**, **i**: Subsarcolemmal membrane myelin-like inclusions and sarcoplasmic localization (marked with arrows). Magnification ×23,000. **g** Area of muscle fiber myolysis with accumulation of large vacuolated mitochondria. Magnification ×17,000. **h** Degenerating muscle fibers. Myofibril thinning and splitting. Multiple vacuolization. Swelling and loss of plasma membrane integrity. Decreased or absent glycogen content. Magnification ×14000

In subsarcolemmal areas and between myofibrils, clusters of osmiophilic granular material were determined (Fig. 6).

## Discussion

Analysis of the present case of combined inheritance of variants in the *DYSF* and *SIL1* genes allowed us to conclude that the patient had signs of congenital dysferlinopathy and Marinesco–Sjögren syndrome, mutually potentiating the involvement of the skeletal muscles. Clinical signs, including postnatal hypotonia at 2 months, delay in all elements of motor development, and weakness of the flexors of the neck and proximal parts of the lower limbs, correspond to the previously described manifestations of the congenital phenotype of dysferlinopathy in two siblings (5 and 2 years old) from a Spanish family [8] and two (14 and 11 years old) from a Turkish family [9] with a similar homozygous frameshift *DYSF* variant, NM_001130987.2;c.2779delG (p.(Ala927LeufsX21)). In addition, weakness in the muscles of the shoulder girdle was observed in the described case and in the siblings of the Turkish family [9]. Thus, muscle weakness in all cases corresponded to the limb-girdle muscle dystrophy (LGMD) pattern, with involvement of the neck flexors and preservation of normal tendon reflexes. Minimal hypotrophy of the gastrocnemius muscles was observed in our case, whereas in a report by Paradas (2008), the presented MRI data reflected the presence of relative hypertrophy of the medial heads of the gastrocnemius muscles in the 5-year-old Spanish sibling [8]. In the patient described by us and the siblings from the Spanish family, joint contractures were not detected, whereas Ceyhan-Birsoy (2015) described contractures of the hip and ankle joints from the age of 3 years [8, 9].

In the case described here, the delay in motor development was also due to the presence of Marinesco–-Sjögren syndrome (MSS), in which patients could achieve the skill of sitting independently by the age of 4 years and walking by the age of ≥ 6 years [10]. The postnatal hypotension observed in patients with congenital dysferlinopathy is transient and minimal in severity, whereas most patients with MSS experience permanent hypotension [11]. In our study, no growth retardation was observed, but the body weight-to-height ratio was reduced [10, 11].

Myopathic syndrome in MSS, observed in 98% of cases [10, 11], is characterized by predominant weakness in the proximal parts of the limbs (i.e., LGMD pattern) and loss of ambulatory status at the age of 13–28 years (mean 17.4 ± 6.3) years [19]. Myopathy in MSS is associated with the cerebellar syndrome [11].

Typical cerebellar syndromes in MSS include truncal ataxia and minimal intention tremor with dysmetria of the upper limbs [11].

In addition, typical ophthalmological signs of MSS were observed in the clinical status of the patients, particularly convergent strabismus (51% of cases) and nystagmus (45% of cases) [10].

The CK level at the age of 1.6 years was increased to 353 U/L (2.4×N), in contrast to the normal CK values ​​in the siblings of the Spanish family, and at the same time was lower than that in the 2-year-old sibling from the Turkish family with a more severe course of congenital dysferlinopathy (728 U/L: 4.8×N) [8, 9]. In previously described cases, a significant increase in CK level by 8.4–25×N was observed after 3 years of age [8, 9].

The combination of dysferlinopathy in our case with MSS did not lead to a significant increase in CK, because even isolated SSM is characterized by an increase in CK to 377 ± 159 U/L in 70% of cases [10]. However, in some cases, an increase in CK levels up to 2000 U/L was noted [19] (Table 2).

**Table 2:** Comparison of previously described congenital dysferlinopathy phenotypes

Signs	C. Paradas, 2008, [8]	C. Paradas, 2008, [8]	Ceyhan-Birsoy O., [9]	Ceyhan-Birsoy, O. [9]	Bardakov S., this report (2026)
Origin of the patient	5 y.o., (m) Spanish	2 y.o., (f) Spanish	14 y.o., (f) Turkish	11 y.o., (m) Turkish	1,6 y.o. (m) Uzbek
Variants in *DYSF*	c.2779delG (p.(Ala927LeufsX21)) [=]				c.1000 C > T (p.(Arg334Trp)); c.518 C > T (p.(Thr173Met));
Other genes	No				*SIL1*: c.178G > T ( p.(Glu60Ter)) [=]
Postnatal hypotonia	Congenital	Congenital, weak cry	Since 4 m	Since 4 m	Since 4 m
Delayed motor development	+	+	+	+	+
Sitting without support	N/A	N/A	8 m	9 m	No
Walking, months	19	21	30	30	No
Loss of walking	No	No	From 8 y.o.	No	n/a
Type of gait disorder	Trendelenburg				No
Climbing the stairs	Difficult	Difficult	No	Difficult	No
Getting up from the floor	Gower’s sign	Cannot stand up without help	Gower’s sign; since 8 y.o. cannot stand up;	Since 11 y.o. cannot stand up;	No
Neck flexors, points	Weakness since 2 m. Since 5 y.o. – 4	From birth. Since 2 y.o. – 2	From birth. Since 11 y.o. – 4	n/a	From birth – 2
Shoulder abduction, points	Normal	Normal	Since 11 y.o. – 3+	n/a	4
Hip flexion, points	3	2	Since 11 y.o. – 2	n/a	4
Leg flexion, points	n/a	n/a	Since 11 y.o. – 2	n/a	5
Standing on tiptoes	Normal	Normal	n/a	n/a	Normal
Tendon reflexes	Normal	Normal	Normal	Normal	Normal
Contractures	n/a	n/a	Since 3 years in the hip and ankle joints	Since 3 years in the ankle joints	No
CK, U/L	Up to 3 years – 100–200; After 3 years – 1262	At 2 years – 241	At 2 years – 728; at 6 years – 4909	At 3 y.o. – 3800; at 4 y.o. – 3747	At 1,6 y.o. – 353
Body weight	Normal	Normal	n/a	n/a	Decreased
MRI	At 5 years: no fatty infiltration or muscle hypotrophy; Muscle edema in the posterior group of thighs and medial gastrocnemius	At 2 y.o.: Normal	n/a	n/a	At 1.6 y.o.: minimal fatty infiltration and hypotrophy of the medial and posterior muscle groups of the thighs, calf muscles; pronounced edema in the posterior muscle group of the legs
Biopsy (light microscopy)	Variability of the MF size; Regeneration (central nuclei); single necrotic MF; Minimal fibrosis of the endomysium and perimysium; no inflammatory infiltrate	n/a	Variability of the MF size; Regeneration (central nuclei); single necrotic MF; Fibrosis and lipomatosis of the endomysium	No	No variability in the MF size; Single necrotic MF; Moderate fibrosis and lipomatosis of endomysium and perimysium; Single CD4+; CD8+, CD68+
IHC with antibodies to dysferlin	Absent	n/a	Absent	n/a	Absent in 99% MF

*m* male, *f* female, *n/a* data not available, *MF* Muscle fibers

MRI signs, represented by pronounced edematous changes in the gastrocnemius muscles in the 1.6 year-old, correspond to the previously presented MRI characteristics of a 5-year-old patient from a Spanish family, whereas in the 2-year-old sibling, MRI changes were not detected [8]. In contrast to the previously described cases, we detected minimal fatty infiltration in the medial and posterior muscle groups of the thigh and gastrocnemius muscles at 1.6 l. Furthermore, pronounced edematous changes in the posterior muscles of the lower legs are not accompanied by a decrease in the strength of the flexors of the feet [8].

MRI signs of MSS include typical atrophy of the vermis and cerebellar hemispheres [11]. In 2006, Mahjneh et al. described in detail the CT pattern of muscle damage in MSS, which was characterized by predominant fat replacement of the muscles of the pelvic girdle, lower limbs, and, to a lesser extent, the shoulder girdle and trunk. Among the muscles of the shoulder girdle and trunk, predominant damage was observed in the m. serratus anterior, m. latissimus dorsi, m. rhomboideus, m. multifidus, and m. iliopsoas [20]. Among the pelvic muscles, the m. gluteus maximus et medius and tensor fasciae latae are the most affected, whereas at the level of the hips, the m. vastus medialis et intermedius, adductor muscles, and m. semimembranosus are more involved in total fat replacement [20, 21]. Using CT and MRI at the level of the shins, a pattern of predominant damage to the posterior group and peroneal muscles, similar to dysferlinopathy, was established [20, 21].

The case described by us and all previously presented patients with congenital dysferlinopathy were registered in consanguineous families [8, 9]. Moreover, in both the previously described families, a similar homozygous *DYSF* frameshift variant c.2779delG (p.(Ala927LeufsX21)) was revealed, which was also observed among Libyan Jews with the LGMD phenotype and Miyoshi myopathy with an average age of onset of 21.6 ± 6.5 years (10–30 years) [22] and in an Iranian patient with a pseudometabolic phenotype (painful calf swelling without atrophy or weakness, exercise intolerance, elevated CK, and episodes of rhabdomyolysis) [23]. Dysferlin expression in the skeletal muscles was completely absent [8]. In our case, two heterozygous *DYSF* missense variants classified as variants of uncertain significance (VUS) were identified: NM_001130987.2:c.1000 C > T (p.Arg334Trp) and NM_001130987.2:c.518 C > T (p.Thr173Met), one of which (c.1000 C > T) likely led to damage to the consensus-splicing site. However, IHC reactions with antibodies against dysferlin revealed a membrane pattern in only 1% of the muscle fibers.

The cause of MSS development was a previously described homozygous *SIL1* variant, NM_022464.5:c.178G > T (p.Glu60Ter) [11, 24].

Possibly, the congenital phenotype is due to the influence of modifier genes. In our case, variants in the *SIL1* gene could have had a similar effect.

Muscle biopsy was characterized by minimal signs of myopathic processes, such as in previously reported cases [8, 9], which confirmed the development of pronounced degenerative changes only after the onset of active independent walking and corresponding increase in the level of CK. The combination of congenital dysferlinopathy with SMS did not lead to an increase in the severity of the myopathic process, which can be of varying degrees of severity in this syndrome [11].

IHC reaction with antibodies against dysferlin revealed a membrane pattern in only 1% of muscle fibers, whereas in patients carrying the *DYSF* variant c.2779delG (p.(Ala927LeufsX21)), dysferlin expression was completely absent [8, 9, 22].

The previously described predominance of type 1 muscle fibers in MSS in the described case was not revealed when typing muscle fibers using myosin heavy chains (44 (39; 50)% slow fibers; 54 (49; 61)% fast fibers) [25].

At the ultrastructural level, characteristic signs of dysferlinopathy were revealed: compaction and fragmentation of the basement membrane [26, 27]; folding and focal ruptures of the sarcolemma [26–28]; anomalies in the structure and distribution of mitochondria [26, 28]; vesicles in the area of ​​sarcolemma defects [26–28]; fine-granular contents in the cytoplasm [29]; thinning, splitting, and focal lysis of myofibrils [26, 28–30]; Z-lines “streaming” [28]; a decrease in the number of T-tubules and sarcotubular cisterns; nuclear polymorphism, folding, and partial lysis of the nucleolemma; and changes in the distribution of chromatin [29].

Typical signs of myopathy in MSS observed were: membranous structures (“myeloid figures”) around the nuclei and in subsarcolemmal areas [11, 12], perinuclear vacuoles, karyopyknosis, accumulation of osmeophilic granular material in the sarcoplasm, and changes in the structure of mitochondria [12, 31]. Notably, the ultrastructural signs of MSS were minimally expressed in our patient. It has been previously reported that in one of the described siblings with an identical variant, no specific signs of MSS were detected during histopathological examination of the skeletal muscles [24].

*SIL1* encodes a nucleotide exchange factor for HSPA5 chaperone protein family member 70, which plays a vital role in regulating endoplasmic reticulum function. *SIL1* defects disrupt the SIL1-HSPA5 interaction, leading to the accumulation of unfolded and/or misfolded proteins in the endoplasmic reticulum, and may induce signaling pathways that lead to cellular degeneration [24].

Possibly, the presence of the identified missense variants in the *DYSF* gene would not have caused the development of the congenital phenotype, but the presence of a variant in the *SIL1* gene probably additionally affected the efficiency of sarcolemma repair involving dysferlin due to the disruption of endoplasmic reticulum function, leading to the observed phenotype.

## Conclusion

This clinical case is an example of the cosegregation of two hereditary diseases with skeletal muscle damage, mutually modifying the clinical phenotype, which requires complex diagnostics with the assessment of ultrastructural changes.

## Data Availability

All data supporting the findings of this study are available within the paper and its Supplementary Information. Information on the identified variants is contained in the ClinVar of the National Center for Biotechnology Information (Accession: VCV004277231.1; VCV004526590.1; VCV001360207.5).
